# Dioscoreae Rhizoma starch improves chronic diarrhea by regulating the gut microbiotas and fecal metabolome in rats

**DOI:** 10.1002/fsn3.3567

**Published:** 2023-08-10

**Authors:** Qing Zhang, Xu Zhang, Qing Wang, Suiqing Chen

**Affiliations:** ^1^ School of Pharmacy Henan University of Chinese Medicine Zhengzhou China

**Keywords:** 16s rRNA, chronic diarrhea, *Dioscorea opposita* Thunb., metabolomics, starch

## Abstract

Chinese yam (*Dioscorea opposite* Thunb.) has been used as food and medicine to treat diarrhea for thousands of years. This article aimed to elucidate the potential mechanism of Dioscoreae Rhizoma starch in alleviating chronic diarrhea induced by rhubarb based on gut microbiotas and fecal metabolome. The administration of the Dioscoreae Rhizoma aqueous extracts, crude polysaccharides, and starch could improve diarrhea and alleviate intestinal injury in chronic diarrhea rats. The Dioscoreae Rhizoma starch displayed the most apparent effect on regulating intestinal microbiotas by increasing the abundance and diversity of microbiotas. At the genus level, there were 17 changed intestinal microbiotas in model rats, and the treatment with Dioscoreae Rhizoma starch regulated 11 microbiotas. Metabolomics analysis revealed that Dioscoreae Rhizoma starch could regulate abnormal fecal metabolites to alleviate diarrhea, and these metabolites are involved in phenylalanine, tyrosine, and tryptophan biosynthesis; tyrosine metabolism; vitamin B6 metabolism; and purine metabolism. This study will contribute to the further research and development of Dioscoreae Rhizoma starch.

## Graphical abstract

Yam starch alleviated the symptoms of chronic diarrhea in rats, and its effect is better than that of the water extract and crude polysaccharides of yam; it improved intestinal injury of rats with chronic diarrhea; it increased the abundance and diversity of intestinal microbiotas in rats with chronic diarrhea and regulated the abnormal intestinal microbiotas; and it displayed a beneficial role by regulating phenylalanine, tyrosine, and tryptophan biosynthesis; tyrosine metabolism; vitamin B6 metabolism; and purine metabolism.

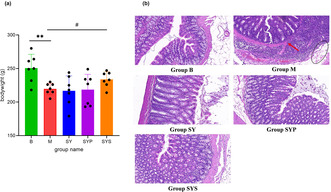

## 1 INTRODUCTION

*Dioscorea opposita* Thunb., also called Chinese yam, is a commonly used medicinal and food homologous Chinese medicine, and it was first described in *Shennong's Classic of Materia Medica* with health benefits. Dioscoreae Rhizoma (dried yam) possesses the effect of improving the functions of the spleen, stomach, lungs, and kidneys. It is always used for treating inappetence, long‐term diarrhea, asthma and cough, spermatorrhea, morbid leukorrhea, frequent urination, and diabetes in the clinic (Chinese Pharmacopoeia Commission, 2020). Among these effects, the invigorating spleen effect is the main one. The main chemical components of Dioscoreae Rhizoma include allantoin, fatty acids, amino acids, proteins, nonstarch polysaccharides, and starch (Chen et al., 2020). Among these components, allantoin (Da Silva et al., 2018; Delibas et al., 2021) and nonstarch polysaccharides (Huang, Xie, et al., 2020) were the focus of many pharmacological studies, while few studies paid attention to the pharmacological effects of Dioscoreae Rhizoma starch, which is the most abundant component in Dioscoreae Rhizoma.

Starch is an essential source of energy for the human body. Resistant starch is one type that can hardly be digested in the stomach and small intestine but can be degraded by intestinal microbiotas in the colon to produce short‐chain fatty acids and other metabolites (Zhu et al., 2020). These metabolites can play a pharmacological effect by regulating intestinal microbiotas and bile acids secreted by the host (Jiang et al., 2020; Lei et al., 2020). Resistant starch has low water holding capacity, no special odor, and good food processing performance. Starch is the main component in Dioscoreae Rhizoma, with a content of 60%–80% (Jiang et al., 2012). The proportion of resistant starch in Dioscoreae Rhizoma starch was also relatively high, ranging from 66.6% to 88.5% (Chen et al., 2016). The high content of resistant starch may display its potential to regulate intestinal microbiotas. Dioscoreae Rhizoma is often used in many Chinese patent medicines as a whole powder containing starch, and the efficacy of this starch needs in‐depth research.

Dioscoreae Rhizoma starch is commonly used to treat chronic diarrhea (Liu, 2017; Zhou & Zhou, 2014). However, only some studies on the antidiarrheal effect and the material basis of Dioscoreae Rhizoma in the treatment of chronic diarrhea still need to be discovered. A systematic study on the pharmacological effect of Dioscoreae Rhizoma in the treatment of chronic diarrhea helps find the efficacy‐related material basis of it.

Chronic diarrhea is a common symptom of many gastrointestinal disorders, such as ulcerative colitis and irritable bowel syndrome. The occurrence and development of chronic diarrhea are closely related to the disorder of intestinal microbiomes, and 16S rRNA sequencing technology is a common technique for studying the changes in intestinal microbiomes (Tong et al., 2022; Zhang et al., 2022). Metabolomics was used to study the changes in endogenous metabolites to clarify the pathogenesis of diseases and the potential therapeutic mechanism of drugs (Liang et al., 2022). In this study, a chronic diarrhea rat model was used to evaluate the effect of relieving diarrhea of different components from Dioscoreae Rhizoma, and the 16S rRNA sequencing method and the ultra‐high performance liquid chromatography coupled quadrupole time‐of‐flight mass spectrometry (UPLC‐Q‐TOF/MS)‐based metabolomics were used to explore potential mechanisms. As far as we know, we are the first to study the mechanism of Dioscoreae Rhizoma components in alleviating chronic diarrhea based on metabolomics and gut microbiota.

## 2 MATERIALS AND METHODS

### 2.1 Materials and reagents

The decoction pieces of Dioscoreae Rhizoma and Rhei Radix Et Rhizoma (rhubarb) were bought from Zhangzhongjing pharmacy (Zhengzhou, China), and these pieces were confirmed by Professor Suiqing Chen from the Henan University of Chinese Medicine to ensure their authenticity.

High‐performance liquid chromatography‐grade methanol, acetonitrile, and formic acid were purchased from Thermo Fisher Technology Co., Ltd. (MA, USA). Other analytical pure reagents were purchased from Tianjin Fuyu Fine Chemical Co., Ltd. (Tianjin, China).

### 2.2 Preparation of the herb extracts

#### 2.2.1 The aqueous extract of the Dioscoreae Rhizoma (SY)

The preparation of the Dioscoreae Rhizoma aqueous extract was carried out according to the methods reported by Zeng et al. (2019) with minor modifications: Ten folds volume of distilled water was added to the flask containing dried yam slices and the mixture was refluxed for 1 h at 100°C after soaking for 20 min. The filtrate was collected and the residue was refluxed once again. The filtrates were mixed and concentrated to eightfold the volume of dried yam slices by a rotary evaporator (RE‐2000A, Tokyo Rikakikai Co., Ltd.) at 50°C and then dried in a freeze dryer (FDU‐1200, Tokyo Rikakikai Co., Ltd.).

#### 2.2.2 The crude polysaccharides of the Dioscoreae Rhizoma (SYP)

The Dioscoreae Rhizoma crude polysaccharides, nonstarch polysaccharides, were prepared according to the methods reported by Wang et al. (2021) with minor modifications. Ten times the volume of anhydrous ethanol was added to the flask containing the dried yam slices and the reflux was carried out at 85°C for 2 h. Then, 10 times the volume of 80% ethanol was added to the residue obtained after filtration that was carried out after refluxing for 2 h. For warm soaking at 90°C for 1.5 h, 10 times the volume of distilled water was added to the filter residue and the mixture was filtered. The warm soaking was repeated once again after filtration. The mixed filtrates were concentrated to eight folds of the volume with a rotary evaporator at 50°C. The anhydrous ethanol was added to the concentrated solution, with the final concentration of ethanol being 80%. The mixture was stored in a refrigerator at 4°C for 24 h and then filtered. The obtained filter residue was thoroughly washed with anhydrous ethanol and acetone. Finally, the residue was stored in a −80°C refrigerator and dried in a freeze dryer.

#### 2.2.3 Dioscoreae Rhizoma starch (SYS)

Dioscoreae Rhizoma starch was prepared according to the method reported by Huang et al. (2021) with minor modification. The screened Dioscoreae Rhizoma powder was added with a 10‐fold volume of anhydrous ethanol stirred for 2 h by a magnetic stirrer at 40°C and then filtered. The 10‐fold volume of 0.03% sodium hydroxide solution was added to the obtained filter residue and then stirred for 2 h by a magnetic stirrer (repeated twice). The supernatant was then removed after sufficient precipitation. The precipitate was washed with distilled water 5–8 times and the mixed solution was adjusted to pH 7 with 0.1 mol/L hydrochloric acid solution. The supernatant was then removed after sufficient precipitation. A 10‐fold volume of anhydrous ethanol was added to the residue and stirred by a magnetic stirrer for 1 h. The mixed solution was filtered, stored in a −80°C refrigerator, and dried in a freeze dryer after the ethanol evaporated. According to the method described previously (Chen et al., 2016), the content of resistant starch in Dioscoreae Rhizoma was determined to be 70.7%.

#### 2.2.4 The water extract of rhubarb

Ten times the volume of distilled water was added to the flask containing slices of rhubarb and then soaked at 50°C for 1 h, the soaking was repeated once after filtration. The two filtrates were combined and concentrated to 1 g/mL by a rotary evaporator at 40°C.

### 2.3 Animal experiments

Thirty‐five male SD rats (160 ± 10 g) were purchased from Shandong Jinan Pengyue Experimental Animal Breeding Co., Ltd. Rats were raised in the Animal Experimental Center of the Henan University of Chinese Medicine. The rats were kept in the animal house with 12 h of light and 12 h darkness. Moreover, the condition temperature was 23 ± 2°C and the humidity was 60 ± 5%. Animal experiments were approved by the Animal Ethics Committee of the Henan University of Chinese Medicine (NO: DWLL202103192). All animal experiments followed the National Institutes of Health guide for the care and use of laboratory animals. After 1 week of adaptation, rats were randomly divided into five groups, including blank control (B) group, model (M) group, Dioscoreae Rhizoma aqueous extract (SY) group, Dioscoreae Rhizoma crude polysaccharide (SYP) group, and Dioscoreae Rhizoma starch (SYS) group, with seven rats in each group. A chronic diarrhea model was prepared by intragastric administration of rhubarb extract. During the modeling process, rats in the M, SY, SYP, and SYS groups were administered the water extract of rhubarb at a dose of 10 mL/kg for 7 days, twice a day, at 8:00 a.m. and 4:00 p.m. (Shi et al., 2020; Yang et al., 2020). During this period, the rats in the B group were given the same volume of distilled water. From the eighth day, rats in SY, SYP, and SYS were treated with the Dioscoreae Rhizoma aqueous extract (0.6 g/kg), crude polysaccharide (0.3 g/kg), and starch (2 g/kg) twice a day, respectively. The rats in the B group and the M group were given the same volume of distilled water. The treatment time lasted 7 days. During the experiment, the daily activities, hair status, mental status, and fecal status of rats in each group were observed. At the end of the experiment, rats were fasted for 12 h and had free access to water. Moreover, the feces of the rats were collected during this period, and the diarrhea rate was also calculated.

The rats were anesthetized with 10% pentobarbital sodium. The colon content and colon tissue of the animals were collected. The colon contents were stored in a sterile sampling tube, then quickly frozen in liquid nitrogen, and stored in a −80°C refrigerator to analyze intestinal microbiotas. Four centimeters from the anus, fresh colon tissues were collected and immersed in 4% paraformaldehyde for histopathological analysis. After adequately storing in paraformaldehyde, the colon tissues were dehydrated, embedded in paraffin, and sliced into 4 μm sections. After hydrated and deparaffinized, the paraffin sections were stained with hematoxylin and eosin. Changes in colon histological sections were observed under an optical microscope (Xu et al., 2022).

### 2.4 Gut microbiota analysis

At the end of the experiment, the colon contents of the rats were quickly collected and immediately frozen in liquid nitrogen and stored at −80°C. Gut microbiota analysis was carried out by Shanghai Biotree Biotech Co., Ltd. The main process was carried out as follows: the DNA was extracted using the DNA extraction kit according to the protocols. The concentration and purification of the final DNA were detected by a micro UV–vis spectrophotometer and the DNA quality was detected by 1% agarose gel electrophoresis. The V3–V4 variable regions of the bacteria 16S rRNA gene were amplified by PCR using universal primers (515F and 806R) with barcode sequences for multiplexing reads of each sample. Then, the sequencing was performed on an Illumina NovaSeq platform and 250 bp paired end reads were generated. Paired end reads were assigned to samples based on their unique barcode and truncated by cutting off the barcode and primer sequence. Paired end reads were merged using fast length adjustment of short reads (FLASH), a high‐speed and accurate analysis tool designed to merge paired end reads when at least some of the reads overlap the read generated from the opposite end of the same DNA fragment, and the splicing sequences were called raw tags. Quality filtering on the raw tags was performed under specific filtering conditions to obtain the high‐quality clean tag according to the QIIME quality‐controlled process (Wen et al., 2021). The tags were compared with the reference database (Silva database) using the UCHIME algorithm to detect chimera sequences, and then the chimera sequences were removed. The effective tags were finally obtained. In order to study the species composition of each sample, the effective tags of all samples were clustered by OTUs (operational taxonomic units) with 97% identity, and then the sequence of OTUs was annotated.

Alpha diversity is used to analyze the microbial community diversity within the sample. The richness and diversity of microbial communities in the sample can be reflected through the diversity analysis of the single sample. The visualized pictures were drawn by GraphPad prism 9.0 (GraphPad, United States). Beta diversity is the comparative analysis of microbial community composition in different samples. First, according to the species annotation results of all samples and the abundance information of OTUs, the same classification of OTUs information was combined to obtain the species abundance information table. At the same time, the unweighted Unifrac distance is further calculated by using the system occurrence relationship between OTUs. Finally, the differences between different samples (groups) are found through multivariate statistical methods, including principal coordinates analysis (PCoA) and nonmetric multi‐dimensional scaling (NMDS) analysis (Gong et al., 2021). According to the species annotation results, the top 10 species at the phylum level with the highest relative abundance in each sample or group at each taxonomic level were selected and the top 30 species at the genus level with the highest relative abundance were selected to generate the column accumulation diagram of relative abundance (Wen et al., 2021). The 16S rRNA sequencing data are submitted to the NCBI database and the accession number is SUB12505566.

### 2.5 Metabonomics analysis

#### 2.5.1 Sample preparation

The collected rat feces were frozen in a refrigerator at −80°C and then dried with a freeze dryer. Feces (50 mg) were weighed accurately and added with 400 μL of 50% methanol (Han et al., 2019), then homogenized for 5 min. After standing for 10 min, the samples were centrifuged for 15 min (13,000 rpm, 4°C) and the supernatant was transferred into a sample bottle for UPLC‐Q/TOF‐MS injection analysis. Samples (20 μL each) were taken equally and mixed as quality control (QC) samples. After being processed, QC samples were injected to every six samples to test the stability and repeatability of the instrument.

#### 2.5.2 Chromatography separation and mass spectrometry conditions for UPLC‐Q/TOF‐MS analysis

Chromatographic separation was performed on a Waters Acquity UPLC HSS T3 C18 column (2.1 mm × 100 mm, 1.8 μm) using an UHPLC system (Agilent Technologies, Inc.). The mobile phase consisted of solvent A (water containing 0.1% formic acid) and solvent B (acetonitrile), and the chromatographic separation was carried out with gradient elution; the mobile phase gradient program was 0 → 15 min, 3% B → 15% B; 15 → 19 min, 35 %B → 40% B; 19 → 25 min, 40%B → 50% B; 25 → 33 min, 70%B → 90%B, 33 → 40 min, 90%B, and the post run time was 3 min. The column temperature was maintained at 35°C and the injection volume was 1 μL.

Mass spectrometry data were acquired on an Agilent 6546 Q‐TOF/MS (Agilent Technologies, Inc.). The instrument was operated with an electrospray ion (ESI) source in positive and negative modes. The ionization source conditions were as follows: drying gas temperature was 320°C, drying gas flow rate was 9 L/min, nebulizer pressure was 38 psi, sheath gas temperature was 350°C, sheath gas flow rate was 11 L/min, and the fragmentor voltage was 110 V. Capillary voltage and nozzle voltage were different in positive and negative modes, as follows: 3500 V and 500 V in positive mode, 4000 V and 1000 V in negative mode, respectively. The mass scanning range was 50–1100 m/z, and the collision energy was 10 V, 20 V, and 30 V for the target MS/MS analysis.

#### 2.5.3 Strategy for analysis of metabolomics

The raw data were collected by Masshunter (Agilent Technologies, Inc.), then processed by Profinder 10.0 (Agilent Technologies, Inc.), including peak extraction, peak alignment, and retention time correction. The exported data from Profinder 10.0 were normalized and filtered by Mass Profiler Professional 15.0 (MPP, Agilent Technologies, Inc.). One‐way analysis of variance (ANOVA) was used to analyze the differences between different groups, and the difference between every two groups was performed by Student's *t* test. The fold change (FC) was analyzed by MPP. SIMCA‐P 14.1 (Umetrics, Sweden) was used for principal component analysis (PCA) and orthogonal partial least squares discriminant analysis (OPLS‐DA), and permutation testing was used to evaluate the accuracy and reliability of the constructed OPLS‐DA model. The confirmation of the importance of the variables was represented by the variable importance in the projection (VIP). The biomarkers were confirmed based on the candidate compounds with VIP value >1, *p* < .05, and the absolute value of FC >2 (Yu, Liang, et al., 2022).

### 2.6 Statistical analysis

Statistical analysis was performed using SPSS 24.0 software (IBM, USA). Comparison among multiple groups was analyzed using one‐way ANOVA. Data are presented as mean ± standard deviation. The *t* test or nonparametric test was used for comparative analysis between the two different groups. The *p* < .05 was considered statistically significant.

## 3 RESULTS

### 3.1 Behavior observation of rats

**FIGURE 1:**
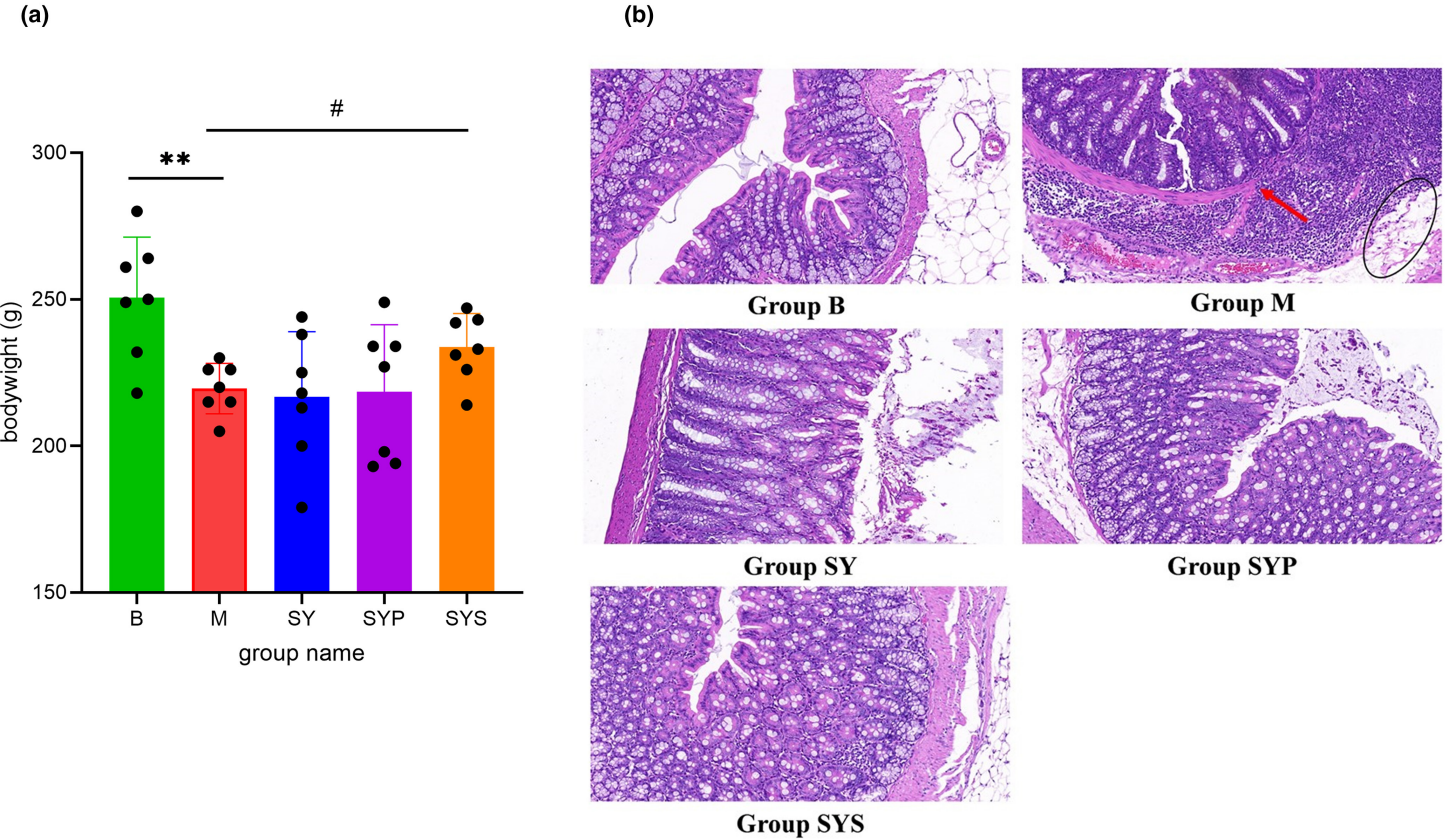
The body weight (a) and the HE staining (b) of colon tissue of rats in each group. **p < .01 compared with group B, #*p* < .05 compared with group M.

During the experiment, rats in the B group displayed a normal mental state, normal activity, spindle‐shaped stool, and shiny hair. The rats in the M group showed less activity, loose stool, filthy anus, withered hair, and skin laxity, with a 100% diarrhea rate. After administration, the diarrhea rates in the SY, SYP, and SYS groups were 42.7%, 28.6%, and 14.3%, respectively. Rats in the SY, SYP, and SYS groups had daily mental status improved, showing increased activity, improved hair appearance, and improved diarrhea. At the end of the experiment, the weight of rats in the SYS group was significantly higher than that in the M group (*p* < .05, shown in Figure 1a), while there was no significant difference between the SY and SYP groups compared with the M group.

### 3.2 Histopathological analysis

Histopathology analysis of colon tissue was carried out by H&E staining. The colonic epithelium of rats in the B group was intact and continuous, without inflammatory infiltration and structural damage. The colonic epithelium injury often occurs in chronic diarrhea rats (Mei et al., 2022). Our results showed that the integrity of the colonic epithelium in the M group was damaged. Visible edema (black circle in Figure 1b) and evident inflammatory cell infiltration (red arrow in Figure 1b) were observed. After treatment with the Dioscoreae Rhizoma aqueous extract, crude polysaccharides, and starch, the infiltration of inflammatory cells and edema in the colon tissue of rats were improved, as shown in Figure 1b.

### 3.3 Gut microbiota analysis

After the quality filtering, 5153 OTUs were determined at the 97% similarity cut‐off and then the sequence was annotated.

#### 3.3.1 Sequencing depth

**FIGURE 2:**
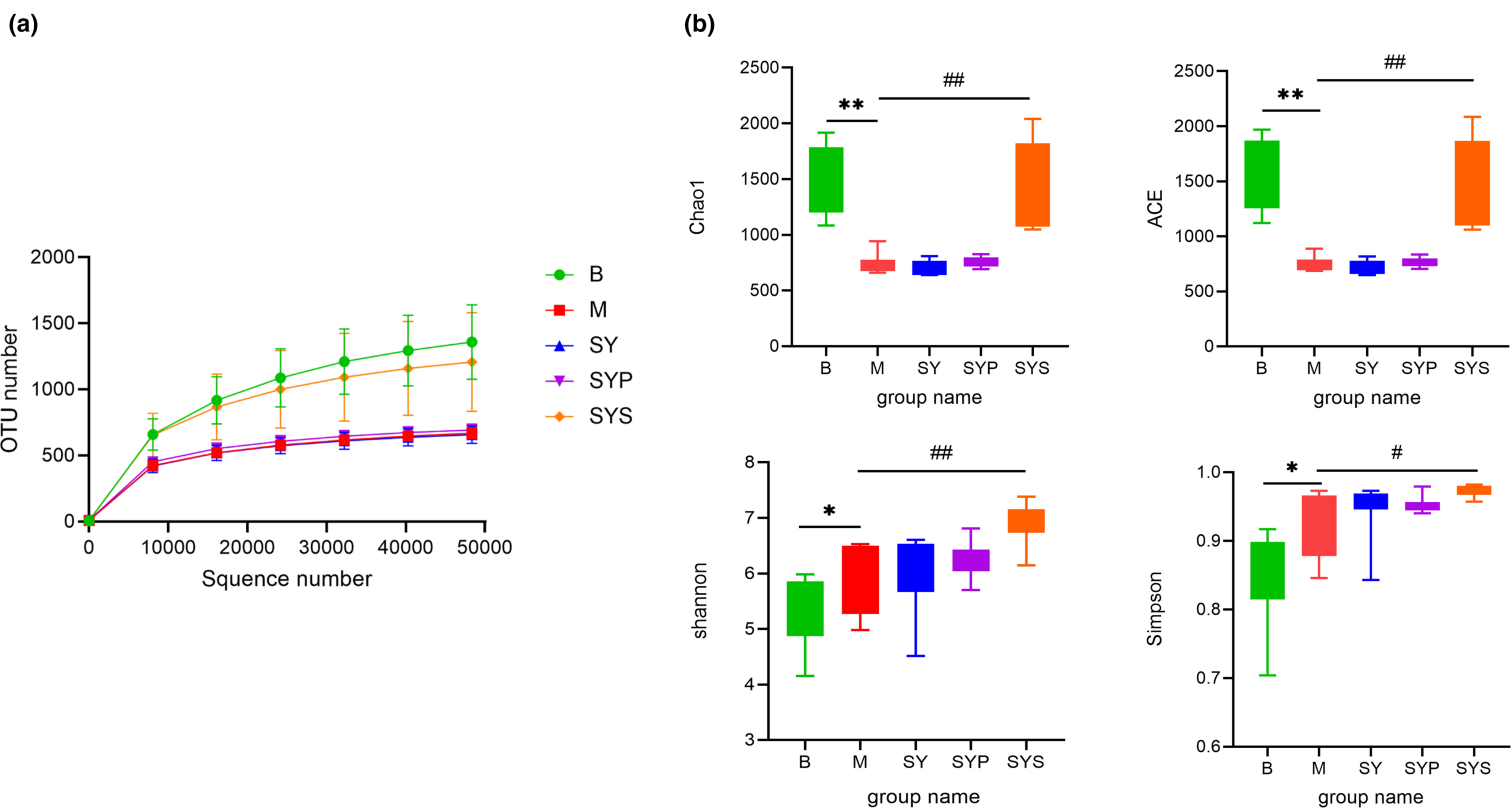
Sequencing depth analysis and α‐diversity analysis (a: rarefaction curve of each group, b: α‐diversity analysis of intestinal flora in each group). *p < .05 compared with group B, **p < .01 compared with group B, #*p* < .05 compared with group M, ##*p* < .01 compared with group M.

The rarefaction curve was constructed by random sampling with the number of sequences and the number of OTUs they could represent (Zhou et al., 2022). The rarefaction curve could also directly reflect the rationality of sequencing data and indirectly reflect the richness of intestinal microbiotas in samples. As shown in Figure 2a, the rarefaction curves of each group tended to be flat, indicating that the sequencing data were reasonable and the sequencing depth has met the analysis requirements.

#### 3.3.2 The gut microbiota diversity in rats

##### The alpha‐diversity analysis

Chao1 and ACE indices were the parameters reflecting the microbial community richness (Chao, 1984; Chao & Lee, 1992). In group M, the richness of intestinal microbiotas was significantly decreased compared with group B. After administration of the aqueous extract and crude polysaccharides of Dioscoreae Rhizoma, the intestinal microbiotas richness did not change significantly, but the richness of intestinal microbiotas was significantly restored after administration of Dioscoreae Rhizoma starch.

Simpson index and Shannon index were the parameters reflecting and microbial community diversity of samples. As shown in Figure 2b, the Simpson index and Shannon index of groups M, SY, SYP, and SYS were higher than those of group B. Among them, the diversity of intestinal microbiotas in group SYS was significantly higher than that in group M. These results indicated that the administration of Dioscoreae Rhizoma starch could increase the diversity and abundance of intestinal microbiotas in chronic diarrhea rats.

##### The beta‐diversity analysis

**FIGURE 3:**
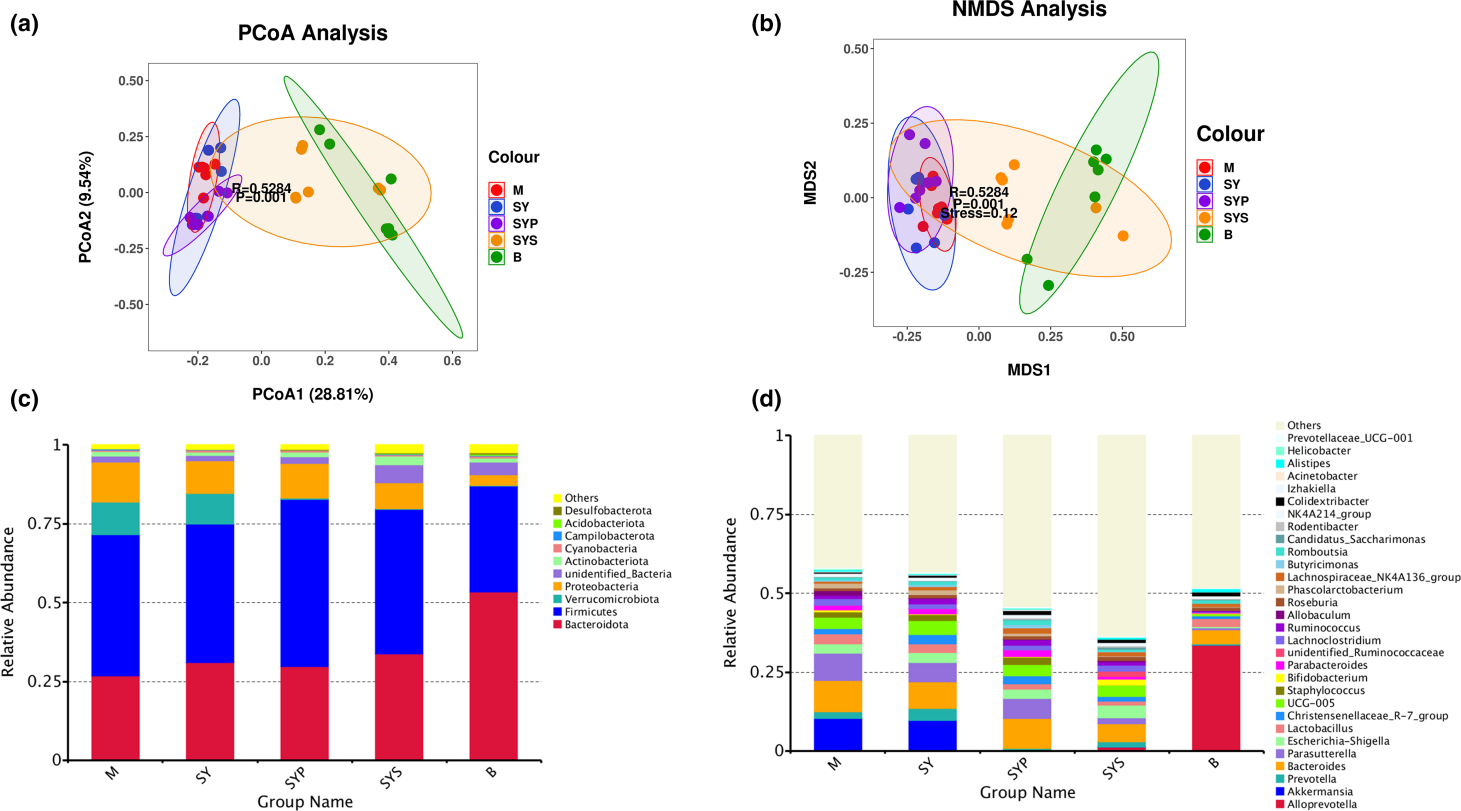
The β‐diversity analysis and histogram of gut microbiotas (a: PCoA analysis, b: NMDS analysis, c: histogram of gut microbiotas distribution at the phylum level, d: histogram of gut microbiotas distribution at the genus level).

The beta‐diversity analysis manifests the differences in microbial composition among animals (Clarke, 1993). PCoA is a commonly used method for beta‐diversity analysis. Bioinformatic analysis and image visualization were performed using the OmicStudio tools (https://www.omicstudio.cn/tool). The PCoA results of intestinal microorganisms in rats of different groups are shown in Figure 3a. The PCoA results show that the contribution rates of the first and second principal components are 28.81% and 9.54%, respectively. The samples in each group were gathered, indicating that the similarity between the groups was good. The M and SYS groups were concentrated on the right side, and the distance was relatively close. In contrast, the other groups were concentrated on the left side, indicating that Dioscoreae Rhizoma starch intervention had a significant indigenous recovery effect on the intestinal microbiotas of chronic diarrhea rats.

NMDS analysis is a nonlinear sorting method that can overcome the shortcomings of linear model analysis, such as PCoA. The sample is reflected in the multidimensional space in the form of points according to the sample's information. The distance between points reflects the difference between different samples. Finally, the two‐dimensional spatial positioning point map of the sample is obtained. Figure 3b also shows that the M, SY, and SYP groups gathered together, and the SYS and B groups are very close, indicating that given Dioscoreae Rhizoma starch can significantly restore intestinal microbiotas in chronic diarrhea rats.

#### 3.3.3 The intestinal microbiotas composition analysis

**FIGURE 4:**
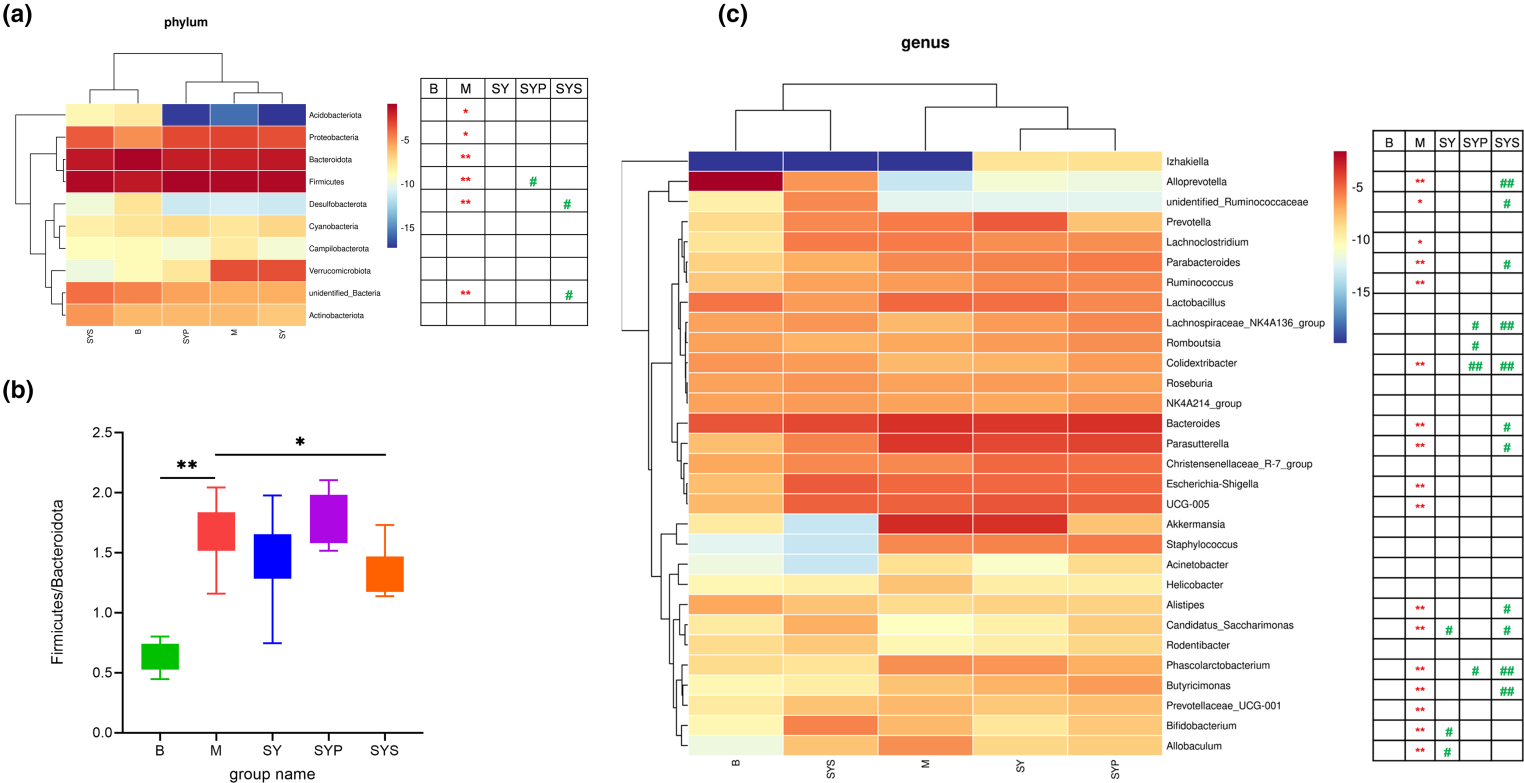
The change of gut microbiotas of rats in each group (a: heatmap of the top 10 gut microbiotas of rats at the phylum level, b: ratio of Firmicutes to Bacteroidetes of rats in each group, c: heatmap of the top 30 gut microbiotas of rats at the genus level). *p < .05 compared with group B, **p < .01 compared with group B; #*p* < .05 compared with group M, ##*p* < .01 compared with group M.

Gut microbiotas at the phylum level mainly consisted of Bacteroidota, Firmicutes, Verrucomicrobiota, Proteobacteria, unidentified bacteria, Actinobacteriota, Cyanobacteria, Campylobacterota, Acidobacteriota, and Desulfobacterota, and these microbiotas represented more than 97% relative abundance (Figure 3c). The relative abundance of Bacteroidota, Firmicutes, Proteobacteria, unidentified bacteria, Acidobacteriota, and Desulfobacterota found in group M significantly differed from group B. The intestinal microbiotas at the phylum level in the SY group displayed no significant change compared with group M, and the relative abundance of Firmicutes in group SYP was higher compared with group M. The relative abundance of unidentified bacteria and Desulfobacterota in the SYS group showed a significantly restored compared to the M group (Figure 4a).

Firmicutes and Bacteroidetes are the two bacteria with the highest relative abundance in the intestinal microbiotas of rats. Their ratios significantly increased in the M group. After treatment with Dioscoreae Rhizoma starch, the ratios are significantly improved (Figure 4b). However, there was no significant change in this ratio in groups SY and SYP.

The relative abundance changes of microbiotas at genus levels were analyzed to further understand the detailed alternations of gut microbiotas. At the genus level, the top 30 relative abundance of rat gut microbiotas include *Alloprevotella*, *Akkermansia*, *Prevotella*, *Bacteroides*, *Parasutterella*, *Escherichia‐Shigella*, *Lactobacillus*, Christensenellaceae_R‐7 group, UCG‐005, *Staphylococcus*, *Bifidobacterium*, *Parabacteroides*, unidentified_Ruminococcaceae, *Lachnoclostridium*, *Ruminococcus*, *Allobaculum*, *Roseburia*, *Phascolarctobacterium*, Lachnospiraceae_NK4A136_group, *Butyricimonas*, Candidatus_Saccharimonas, *Romboutsia*, *Rodentibacter*, NK4A214_group, *Colidextribacter*, *Izhakiella*, *Acinetobacter*, *Alistipes*, *Helicobacter*, and Prevotellaceae_UCG‐001, as shown in Figure 3d.

The abundance of 17 intestinal microbiotas in group M significantly changed, including *Alloprevotella*, *Bacteroides*, *Parasutterella*, *Escherichia*‐*Shigella*, UCG‐005, *Bifidobacterium*, *Parabacteroides*, unidentified_Ruminococcaceae, Lachnoclostridium, *Ruminococcus*, *Allobaculum*, *Phascolarctobacterium*, *Butyricimonas*, Candidatus_Saccharimonas, *Colidextribacter*, *Alistipes*, Prevotellaceae_UCG‐001.

Only three gut microbiotas exhibited a recovery in group SY compared with group M. In group SYP, the relative abundance of four gut microbiotas changed significantly compared with group M (*p* < .05). Compared with M group, 11 microbiotas showed significant change in group SYS (Figure 4c). The results showed that Dioscoreae Rhizoma starch obviously affected the intestinal microbiotas of chronic diarrhea rats and the regulation effect was better than that of the Dioscoreae Rhizoma aqueous extract and crude polysaccharides.

### 3.4 Fecal metabolome analysis

**FIGURE 5:**
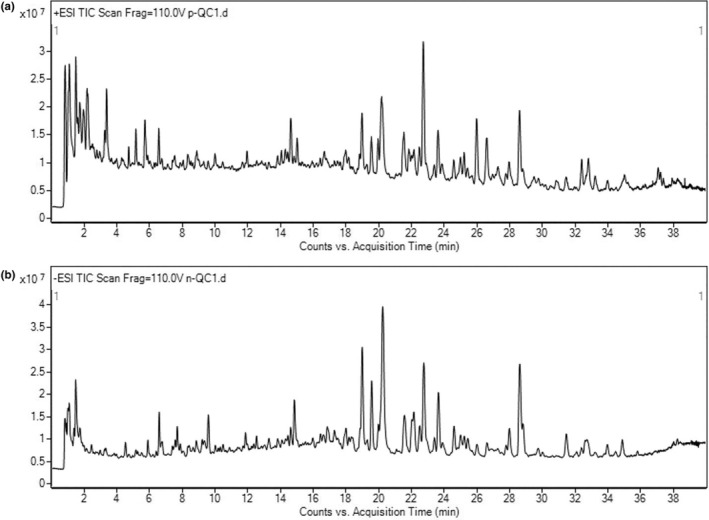
Typical chromatograms of QC samples (a: positive mode, b: negative mode).

In order to further study the potential mechanism of Dioscoreae Rhizoma starch in alleviating diarrhea, an untargeted fecal metabolome analysis was carried out, and the typical total ion chromatograms of the quality control samples (QC) in positive and negative modes are shown in Figure 5.

#### 3.4.1 Principal component analysis

**FIGURE 6:**
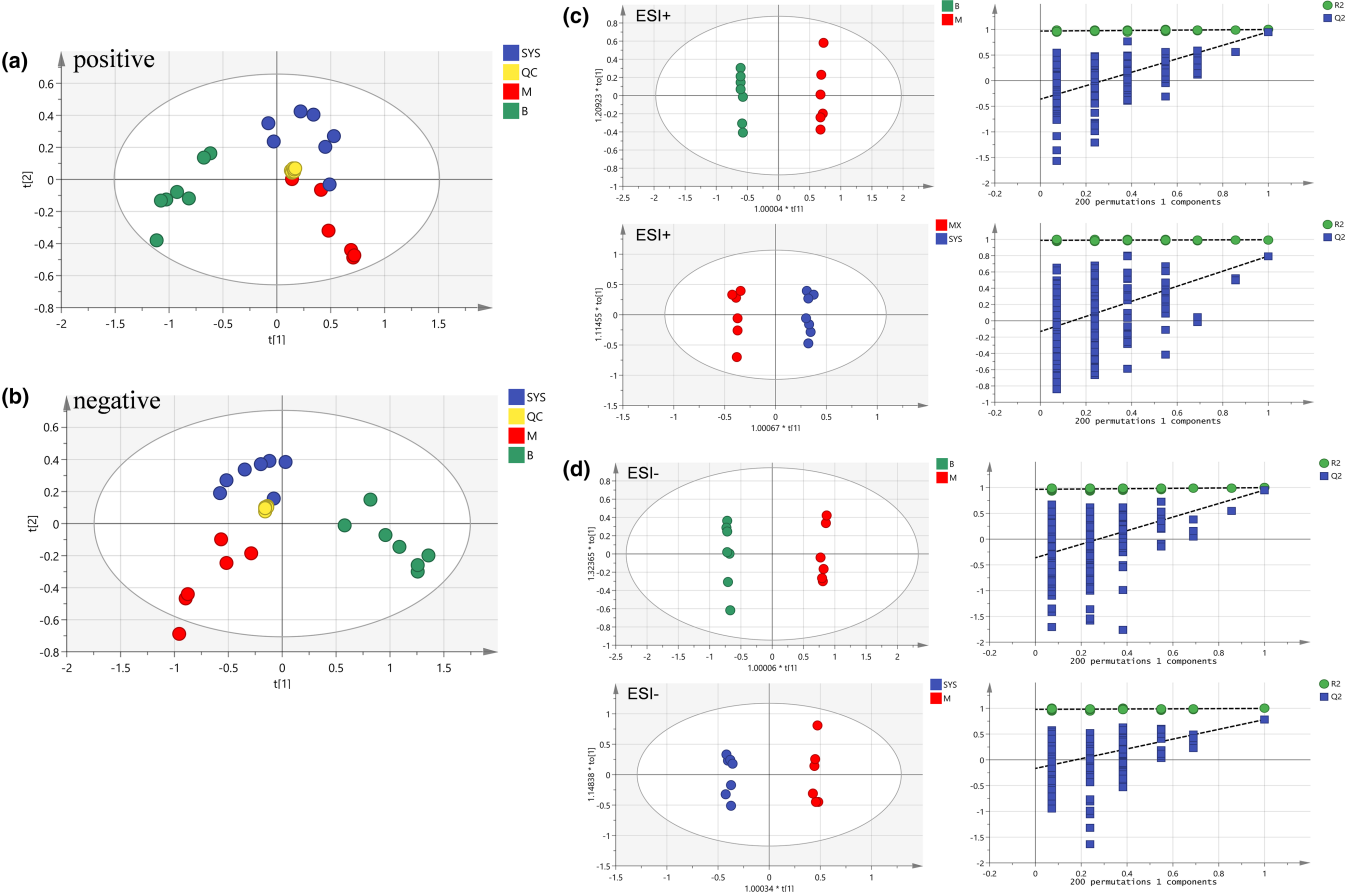
PCA and OPLS‐DA score plots of the metabolites in different groups (PCA score plot in [a] positive and [b] negative ion modes, OPLS‐DA score plot in [c] positive and [d] negative ion modes).

PCA, a typical unsupervised multivariate statistical analysis method, was employed for metabolomics analysis. The sample points in each were clustered (shown in Figure 6a,b), and the PCA scores of QC samples were well gathered in both positive and negative ion modes, indicating that the instrument's operating condition was relatively stable and suitable for the metabolomics analysis.

The PCA scores of groups M and B were entirely separated under the positive and negative ion modes (Figure 6a,b), indicating that the endogenous metabolites of rats in group M were quite different from those in group B. The PCA scores of groups SYS and M were separated under the positive and negative ion modes, and group SYS was close to group B, indicating that Dioscoreae Rhizoma starch could improve the endogenous metabolites change in chronic diarrhea rats.

#### 3.4.2 Orthogonal partial least squares discriminant analysis

OPLS‐DA is a supervised analysis method commonly used to screen potential biomarkers between different groups. Therefore, the OPLS‐DA method was used for further investigating the difference among these groups. The OPLS‐DA scores of the B and M groups (Figure 6c,d) showed that these groups were entirely separated in positive and negative ion modes, indicating that the metabolites in chronic diarrhea rats were different from that in healthy rats. The OPLS‐DA scores also showed that groups SYS and M were separated in positive and negative ion modes. The separation effect of OPLS‐DA was better than that of PCA. Model parameters R^2^Y and Q^2^ are used to evaluate the interpretation rate and the prediction rate of OPLS‐DA, respectively. The closer the model parameters are to 1, the more reliable the models are. The parameter values of each OPLS‐DA model are shown in Table 1. The results indicated that each OPLS‐DA model was relatively reliable and could accurately explain and predict the differences between the two groups. The permutation tests were carried out to analyze whether OPLS‐DA was overfitted, as shown in Figure 6c,d, and the result revealed that OPLS‐DA had not been overfitted, indicating that the analysis results were accurate and reliable.

**TABLE 1:** Model parameters R^2^Y and Q^2^ of OPLS‐DA analysis.

Ion mode	R^2^Y	Q^2^
Positive: B vs. M	0.999	0.953
Positive: SYS vs. M	0.995	0.795
Negative: B vs. M	0.999	0.953
Negative: SYS vs. M	0.997	0.782

### 3.5 Identification of the potential biomarkers

The potential biomarkers were selected based on VIP >1, the absolute value of fold change >2, and *p* < .05 (between groups B and M as well as groups M and SYS; Huang, Ye, et al., 2020). The accurate mass and MS/MS fragments of candidate metabolites matched those of metabolites obtained from METLIN (metlin, scripps.edu) and HMDB (https://hmdb.ca/). The mass tolerance between the measured m/z values and the exact mass of the components was within 5 ppm. If the accurate mass of markers could not match the databases or correlative descriptions were not endogenous, they were excluded from the interested list. Finally, 46 potential biomarkers related to chronic diarrhea were identified, as shown in Table 2 for details. A total of 23 endogenous biomarkers were regulated after being treated with Dioscoreae Rhizoma starch.

**TABLE 2:** The potential fecal biomarkers identified in different groups.

No.	HMDB	Compound	Formula	Ion mode	Mass	Retention time	Group B vs. group M			Group SYS vs. group M		
							Significance	Trend	Fold change	Significance	Trend	Fold change
1	HMDB0001257	Spermidine	C_7_H_19_N_3_	Positive	145.1580	0.858	**	Up	−2.6	—	—	—
2	HMDB0011150	Deoxyhypusine	C_10_H_23_N_3_O_2_	Positive	217.1797	0.986	—	—	—	#	Up	2.2
3	HMDB0006483	d‐Aspartic acid	C_4_H_7_NO_4_	Positive	133.0375	1.008	**	Down	5.5	—	—	—
4	HMDB0002931	*N*‐Acetylserine	C_5_H_9_NO_4_	Positive	147.0530	1.011	**	Down	2.7	—	—	—
5	HMDB0013222	Beta‐Guanidinopropionic acid	C_4_H_9_N_3_O_2_	Positive	131.0697	1.043	**	Up	−3.6	—	—	—
6	HMDB0029944	Ascorbalamic acid	C_9_H_13_NO_8_	Negative	263.0647	1.084	—	—	—	#	Down	−2.6
7	HMDB0001488	Nicotinic acid	C_6_H_5_NO_2_	Positive	123.0320	1.335	**	Down	2.5	—	—	—
8	HMDB0000289	Uric acid	C_5_H_4_N_4_O_3_	Negative	168.0282	1.545	—	—	—	#	Up	2.1
9	HMDB0001431	Pyridoxamine	C_8_H_12_N_2_O_2_	Positive	168.0895	1.805	**	Down	2.7	##	Up	2.3
10	HMDB0000650	d‐2‐Aminobutyric acid	C_4_H_9_NO_2_	Positive	103.0632	2.099	**	Down	3.9	—	—	—
11	HMDB0033977	Methyl acrylate	C_4_H_6_O_2_	Positive	86.0367	3.041	**	Down	3.5	—	—	—
12	HMDB0000678	Isovaleryl glycine	C_7_H_13_NO_3_	Positive	159.0896	3.713	**	Up	−3.1	—	—	—
13	HMDB0028900	Isoleucyl‐alanine	C_9_H_18_N_2_O_3_	Positive	202.1318	3.966	**	Up	−3.7	—	—	—
14	HMDB0029065	Threoninyl‐leucine	C_10_H_20_N_2_O_4_	Positive	232.1423	4.009	**	Up	−3.7	—	—	—
15	HMDB0000291	Vanillylmandelic acid	C_9_H_10_O_5_	Negative	198.0528	4.029	**	Up	−5.1	—	—	—
16	HMDB0031173	6‐Hydroxy‐1H‐indole‐3‐acetamide	C_10_H_10_N_2_O_2_	Positive	190.0742	4.791	—	—	—	#	Up	2.2
17	HMDB0001424	4‐(3‐Pyridyl)‐3‐butenoic acid	C_9_H_9_NO_2_	Negative	163.0632	6.419	**	Down	6.8	#	Up	2.5
18	HMDB0060328	1‐Nitro‐5,6‐dihydroxy‐dihydronaphthalene	C_10_H_9_NO_4_	Positive	207.0531	6.43	**	Down	6.7	#	Up	2.4
19	HMDB0000714	Hippuric acid	C_9_H_9_NO_3_	Positive	179.0578	6.818	**	Down	5.8	##	Up	3.1
20	HMDB0029496	Fukiic acid	C_11_H_12_O_8_	Negative	272.0542	7.246	**	Down	6.1	—	—	—
21	HMDB0034885	Imazamethabenz	C_15_H_18_N_2_O_3_	Positive	274.1316	8.083	**	Down	5.7	##	Up	2.3
22	HMDB0032369	l‐Menthyl acetoacetate	C_14_H_24_O_3_	Positive	240.1724	8.429	**	Down	8.1	#	Up	2
23	HMDB0000792	Sebacic acid	C_10_H_18_O_4_	Negative	202.1203	8.563	**	Down	3.8	##	Up	2.5
24	HMDB0000022	3‐Methoxytyramine	C_9_H_13_NO_2_	Positive	167.0946	8.866	**	Up	−7.2	#	Up	2.6
25	HMDB0028998	Phenylalanyl‐isoleucine	C_15_H_22_N_2_O_3_	Positive	278.1630	8.917	**	Up	−5.7	—	—	—
26	HMDB0062406	4‐Oxo‐1‐(3‐pyridyl)‐1‐butanone	C_9_H_9_NO_2_	Positive	163.0633	9.018	**	Down	8.3	—	—	—
27	HMDB0060684	2‐Propylglutaric acid	C_8_H_14_O_4_	Negative	174.0891	9.391	**	Down	3.7	—	—	—
28	HMDB0033625	(3R,7R)‐1,3,7‐Octanetriol	C_8_H_18_O_3_	Positive	162.1258	10.69	**	Down	2.4	—	—	—
29	HMDB0000158	l‐Tyrosine	C_9_H_11_NO_3_	Positive	181.0739	10.87	**	Down	4.3	##	Up	2.4
30	HMDB0000832	Capryloyl glycine	C_10_H_19_NO_3_	Positive	201.1364	10.912	**	Down	2.5	—	—	—
31	HMDB0034263	Triethyl citrate	C_12_H_20_O_7_	Positive	276.1206	11.061	**	Down	7	—	—	—
32	HMDB0000784	Azelaic acid	C_9_H_16_O_4_	Positive	188.1048	11.874	**	Down	3.1	—	—	—
33	HMDB0012162	4‐Methoxytyramine	C_9_H_13_NO_2_	Positive	167.0946	12.073	**	Up	−2.4	—	—	—
34	HMDB0000413	3‐Hydroxydodecanedioic acid	C_12_H_22_O_5_	Negative	246.1464	12.255	—	—	—	##	Up	2.3
35	HMDB0002341	8‐Iso‐15‐keto‐PGE2	C_20_H_30_O_5_	Negative	350.2090	13.694	**	Down	3.8	—	—	—
36	HMDB0004160	I‐Urobilin	C_33_H_42_N_4_O_6_	Positive	590.3105	14.547	**	Up	−7.5	—	—	—
37	HMDB0030984	5‐Hexyltetrahydro‐2‐oxo‐3‐furancarboxylic acid	C_11_H_18_O_4_	Negative	214.1203	14.584	—	—	—	##	Up	2.8
38	HMDB0000922	Taurallocholic acid	C_26_H_45_NO_7_S	Negative	515.2909	14.622	—	—	—	##	Down	−4.3
39	HMDB0000433	1,3,7,12‐Tetrahydroxycholan‐24‐oic acid	C_24_H_40_O_6_	Negative	424.2820	14.942	**	Down	3.5	—	—	—
40	HMDB0000307	1β,3α,7α,12α‐Tetrahydroxy‐5β‐cholanoic acid	C_24_H_40_O_6_	Negative	424.2821	15.978	—	—	—	##	Up	2.1
41	HMDB0000889	Tauroursocholic acid	C_26_H_45_NO_7_S	Negative	515.2913	16.946	**	Up	−6.3	##	Down	−4.1
42	HMDB0000623	Dodecanedioic acid	C_12_H_22_O_4_	Negative	230.1516	18.327	**	Down	4.4	##	Up	2.6
43	HMDB0000364	3α,6α,7β‐Trihydroxy‐5b‐cholanoic acid	C_24_H_40_O_5_	Negative	408.2873	18.991	—	—	—	#	Up	3
44	HMDB0034295	Phloionolic acid	C_18_H_36_O_5_	Negative	332.2558	19.279	**	Down	3.7	—	—	—
45	HMDB0000626	Deoxycholic acid	C_24_H_40_O_4_	Negative	392.2922	24.033	**	Down	3.9	—	—	—
46	HMDB0000782	Octadecanedioic acid	C_18_H_34_O_4_	Negative	314.2454	24.039	**	Down	4.2	—	—	—
47	HMDB0000503	7α‐Hydroxy‐3‐oxo‐5β‐cholanoic acid	C_24_H_38_O_4_	Negative	390.2767	24.606	**	Down	8.6	—	—	—
48	HMDB0002585	3β,12β‐Dihydroxy‐5β‐cholanoic acid	C_24_H_40_O_4_	Negative	392.2922	25.438	**	Down	5	—	—	—
49	HMDB0011502	PE(15:0/0:0)	C_20_H_42_NO_7_P	Negative	439.2695	28.383	—	—	—	##	Up	5.7
50	HMDB0002536	Isodeoxycholic acid	C_24_H_40_O_4_	Negative	392.2924	28.615	**	Down	4.1			
51	HMDB0011507	PE(18:2(9Z,12Z)/0:0)	C_23_H_44_NO_7_P	Negative	477.2850	29.556	—	—	—	#	Down	−2
52	HMDB0011473	LysoPE(0:0/16:0)	C_21_H_44_NO_7_P	Negative	453.2852	30.805	—	—	—	#	Down	−2.2
53	HMDB0000761	Lithocholic acid	C_24_H_40_O_3_	Negative	376.2972	32.27	**	Down	5.1	##	Up	3.1
54	HMDB0004668	13‐OxoODE	C_18_H_30_O_3_	Negative	294.2192	32.401	**	Down	3.6	—	—	—
55	HMDB0031060	(R)‐2‐Hydroxysterculic acid	C_19_H_34_O_3_	Positive	310.2507	32.902	**	Up	−4.9	—	—	—
56	HMDB0004669	9‐OxoODE	C_18_H_30_O_3_	Negative	294.2192	33.226	**	Down	3.3	—	—	—
57	HMDB0004702	12(13)‐EpOME	C_18_H_32_O_3_	Positive	296.2350	34.506	**	Down	4.5	—	—	—
58	HMDB0031057	2‐Hydroxyhexadecanoic acid	C_16_H_32_O_3_	Negative	272.2348	35.645	**	Down	4.7	—	—	—

*Note*: **p < .01 were compared with group B; #*p* < .05 and ##*p* < .01 were compared with group M.

### 3.6 Metabolic pathway analysis

**FIGURE 7:**
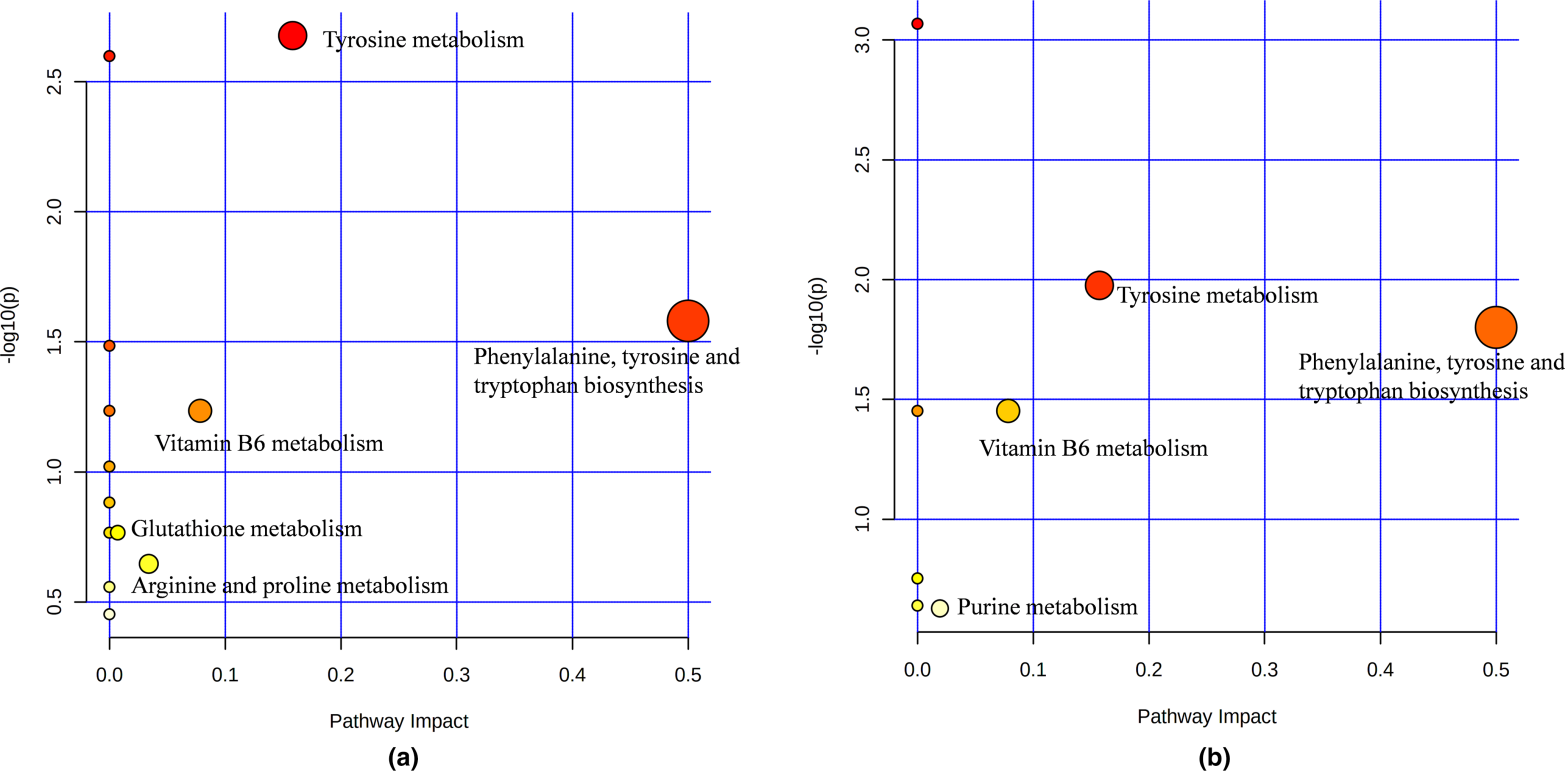
Metabolic pathway analysis of potential biomarkers (a: model group compared with the blank group, b: yam starch group compared with the model group).

The HMDB ID of the potential biomarker was imported into Metaboanalyst 5.0 (http://www.metaboanalyst.ca/) for the metabolic pathway analysis (Pang et al., 2022). The pathway analysis results are shown in Figure 7. Each circle represented a metabolic pathway. The larger area of the circle, the darker color of the circle, and the more influential pathway is. Five critical metabolic pathways were screened with the impact >0, considered to be significantly changed in the chronic diarrhea rats, including phenylalanine, tyrosine, and tryptophan biosynthesis; tyrosine metabolism; vitamin B6 metabolism; arginine and proline metabolism; and glutathione metabolism. Similarly, four critical metabolic pathways were screened with the impact >0, considered significant changes in chronic diarrhea rats after being treated with starch, including phenylalanine, tyrosine, and tryptophan biosynthesis; tyrosine metabolism; vitamin B6 metabolism; and purine metabolism.

### 3.7 Correlation analysis

**FIGURE 8:**
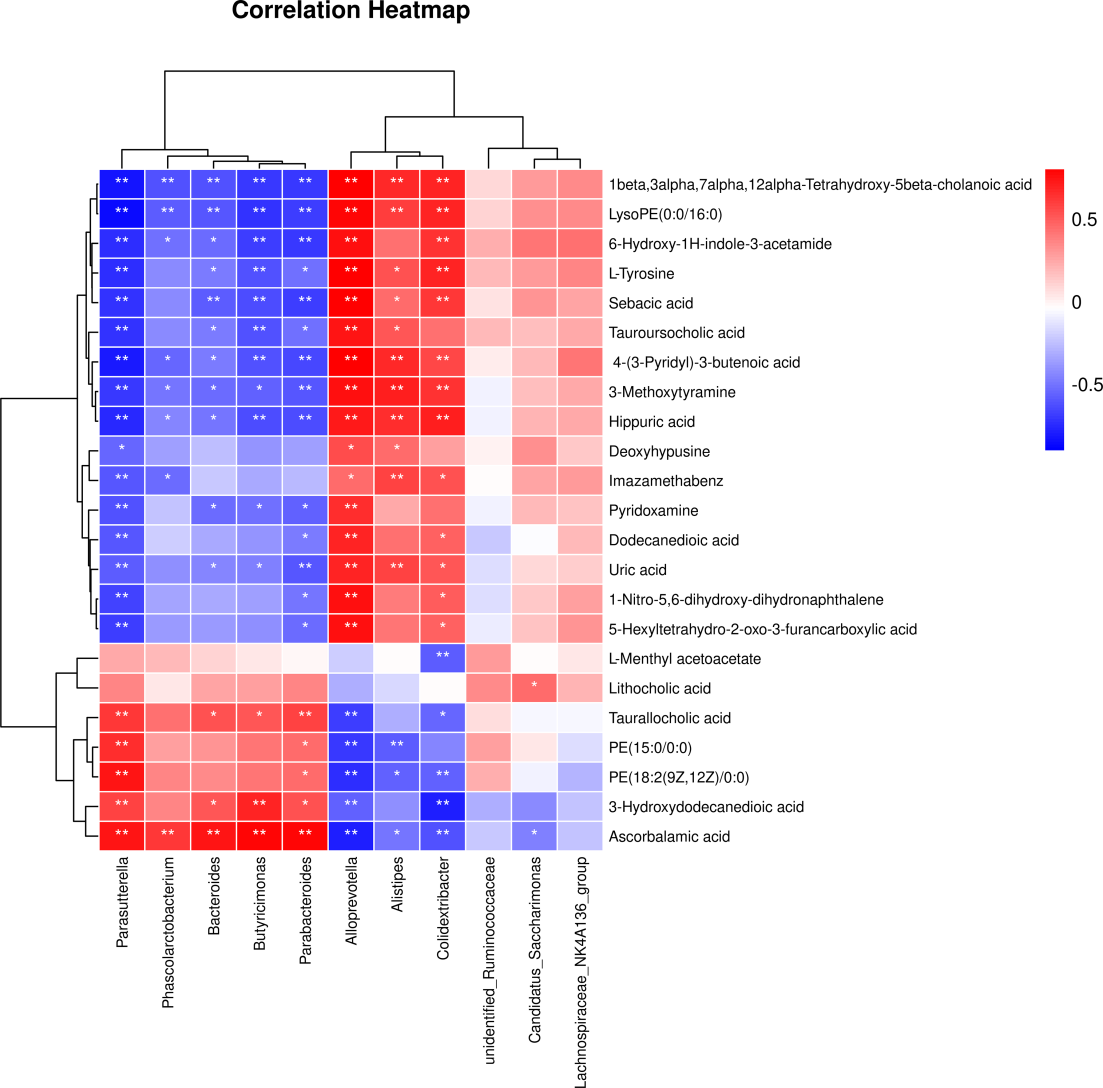
Heatmap of correlation coefficients between the potential metabolic biomarkers and the microbiological biomarkers.

Spearman's correlation analysis was performed to elucidate the interaction between changed metabolites and intestinal microbiotas in group SYS. The correlation analysis between metabolic markers and gut microbiota was analyzed by Omicstudio (https://www.omicstudio.cn/index, *p* < .05 was thought to be a significant correlation, and a heatmap was used to display the correlation coefficients between each pair) and the results were shown in Figure 8. Our results suggested that there is a potentially complex relationship between the gut microbiota and metabolites. Dioscoreae Rhizoma starch may affect the gut microbiota and metabolites in rats to exert a therapeutic effect on chronic diarrhea rats.

For example, *Alloprevotella* and *Parasutterella* are two bacteria closely related to metabolites. These related metabolites include 4‐(3‐pyridyl)‐3‐butenoic acid, 3‐methoxytyramine, uric acid, pyridoxamine, hippuric acid, l‐tyrosine, 6‐hydroxy‐1H‐indole‐3‐acetamide, sebacic acid, 1‐nitro‐5,6‐dihydroxy‐dihydronaphthalene, 5‐hexyltetrahydro‐2‐oxo‐3‐furancarboxylic acid, deoxyhypusine, dodecanedioic acid, 3‐hydroxydodecanedioic acid, ascorbalamic acid, imazamethabenz, 1beta, 3alpha, 7alpha, 12alpha‐Tetrahydroxy‐5beta‐cholanoic acid, PE(15:0/0:0), LysoPE(0:0/16:0), PE(18:2(9Z,12Z)/0:0), tauroursocholic acid, and taurallocholic acid. Interestingly, the correlations of *Alloprevotella* and *Parasutterella* with these metabolites showed opposite trends.

## 4 DISCUSSION

Dioscoreae Rhizoma is a commonly used Chinese medicine with the effect of invigorating the spleen, which can be widely used to treat gastrointestinal diseases such as chronic diarrhea. At present, there are few experimental studies on Dioscoreae Rhizoma for diarrhea treatment. The aqueous extract (Jin et al., 2020) and nonstarch polysaccharides (Liu et al., 2022) of Dioscoreae Rhizoma are the focus of previous studies. Although there are many studies on the physical and chemical characteristics of Dioscoreae Rhizoma starch (Zou et al., 2022), the pharmacological effects of this starch still need to be sufficiently studied. Resistant starch, a type of hard‐to‐digest starch, and its pharmacological effects are gradually discovered. The resistant starch is effective in lowering blood glucose (Zhang et al., 2020) and blood lipids and preventing colon cancer, mainly by regulating the intestinal microbiotas (Latino et al., 2022). The content of resistant starch in Dioscoreae Rhizoma starch is high, and the role of Dioscoreae Rhizoma starch cannot be ignored. In our study, the Dioscoreae Rhizoma starch could alleviate the diarrhea and reduce the intestinal injury of chronic diarrhea rats and improve intestinal inflammation.

Intestinal microbiotas are closely related to the health of the host. They participate in the host's nutritional absorption and immune regulation (Tremaroli & Bäckhed, 2012). The occurrence and development of many diseases are closely related to the disorder of intestinal microbiotas. The pathogenesis of chronic diarrhea is related to the disorder of intestinal microbiotas and the abnormality of endogenous metabolism (Huang, Ye, et al., 2020; Zhang et al., 2022). Our results showed that the features of intestinal microbiotas were significantly changed in chronic diarrhea rats, and the ratio of Firmicutes to Bacteroidetes was significantly increased. Firmicutes and Bacteroidetes are the two most abundant bacteria in the intestinal flora, and the change in their proportion is closely related to the occurrence of many diseases. In rats with chronic diarrhea, there was a marked reduction in the diversity and abundance of intestinal flora, which was significantly suppressed after treatment with Dioscoreae Rhizoma starch. Lachnospiraceae_NK4A136_group exhibits anti‐inflammatory properties and promotes the repair of the intestinal mucosa (Zhang et al., 2021; Zhong et al., 2021). In the model group, the abundance of the bacteria decreased, while the administration of Dioscoreae Rhizoma starch treatment significantly increased the abundance of the bacteria. Similarly, Dioscoreae Rhizoma starch regulated 11 intestinal microbiotas in chronic diarrhea rats at the genus level, and the regulation effect was significantly better than Dioscoreae Rhizoma aqueous extract and crude polysaccharides.

The intestinal flora can affect the host's metabolism through its metabolites and the host's endogenous metabolites can also affect the metabolic response of the intestinal flora. Stool, as a relatively easy‐to‐obtain biological sample, can reflect host and intestinal flora metabolite information (Cheng et al., 2023; Zhang et al., 2023). In order to find the changes in fecal metabolites after the intervention of Dioscoreae Rhizoma starch, LC‐MS‐based metabolomics was carried out. Compared with the rats in group B, 46 potential markers in the fecal samples between groups B and M were changed significantly, and these metabolites were involved in five essential metabolic pathways. After being administered with Dioscoreae Rhizoma starch, 23 metabolites displayed a noticeable change, and these changed metabolites are involved in the four metabolic pathways, including phenylalanine, tyrosine, and tryptophan biosynthesis; tyrosine metabolism; vitamin B6 metabolism; and purine metabolism.

Amino acid metabolism is an essential metabolic process in gastrointestinal‐related diseases (Cao et al., 2022; Zhang et al., 2021). Amino acid is the basic unit of protein, participates in the synthesis of protein and hormones, and plays an essential physiological role in an organism. In our study, the changed amino acid metabolic pathways include phenylalanine, tyrosine, and tryptophan biosynthesis; tyrosine metabolism; and arginine and proline metabolism.

Phenylalanine is one of the essential amino acids in the human body. Many studies have found that phenylalanine metabolism is related to many diseases (Yu, Xu, et al., 2022). Phenylalanine and tyrosine metabolism are closely related to the biochemical processes of neurotransmitter synthesis, glucose metabolism, and fat metabolism. The content of l‐tyrosine in the model rats was significantly lower than that in the blank group, and the amount of l‐tyrosine was significantly reversed after the treatment of Dioscoreae Rhizoma starch. The arginine and proline metabolism involved in arginine may be one of the metabolic pathways of Dioscoreae Rhizoma starch in alleviating diarrheal effect. Downstream metabolites of arginine are polyamines, nitric oxide (NO), and nitrogen oxide (Guo et al., 2022). Studies have shown that NO has a wide range of biochemical properties, can eliminate reactive oxygen species, improve microcirculation, be involved in the synthesis and stabilization of extracellular matrix, increase various metabolic functions of cells, and participate in the repair of hepatocytes as arginine is the only substrates for the synthesis of nitric oxide (NO) in vivo. The upregulation of arginine biosynthesis pathway is helpful for the production of NO. In rats with chronic diarrhea, the level of spermidine, a key metabolite in arginine and glutathione metabolic pathways, was significantly decreased, and the decreasing trend could be reversed after the administration of Dioscoreae Rhizoma starch.

Glutathione plays a decisive role in maintaining protein stability, the structural integrity of the biofilm system, and preventing membrane lipid peroxidation (Chao et al., 2022). Glutathione can also protect hemoglobin from oxidation by hydrogen peroxide and free radicals so that it can continuously and typically play the role of transporting oxygen. Reduced glutathione can directly combine with oxidants such as hydrogen peroxide to generate water and oxidized glutathione and reduce high iron hemoglobin to hemoglobin. Glutathione is a critical substance in the mitochondrial tricarboxylic acid cycle and oxidative respiratory chain. It is the coenzyme and coenzyme of many enzymes and participates in the tricarboxylic acid cycle and glucose metabolism so that the body can obtain energy. In rats of the model group, evident intestinal inflammation appeared, which may be related to the abnormal redox of the host. In rats with chronic diarrhea, glutathione metabolism was abnormal, and the rats recovered after the intervention of Dioscoreae Rhizoma starch.

Purine metabolism is closely related to gastrointestinal‐related diseases and some spleen‐invigorating medicine can play a therapeutic role by regulating purine metabolism (Liu et al., 2020). Purine exists primarily in nucleotide form in the body (Liu et al., 2020). The synthesis of purine nucleotides can be achieved through ab initio synthesis and remediation, which requires a large amount of ATP. ATP is decomposed to produce ADP, and ADP is hydrolyzed to AMP under the action of muscle kinase. AMP generates hypoxanthine nucleotide (IMP) and NH3 under the action of deaminase. IMP can regenerate AMP after amino is obtained, which is also part of the purine nucleotide cycle. Moreover, IMP decomposes into xanthine by hypoxanthine nucleotide dehydrogenase and generates uric acid to discharge in vitro (Crittenden et al., 2018; Li et al., 2022). Our results found that the uric acid content in the Dioscoreae Rhizoma starch group was significantly increased compared to the model group, indicating that the upregulation of purine metabolism may be one of the mechanisms of the protective effect of Dioscoreae Rhizoma starch.

Vitamin B6 metabolism is related to many physiological processes of the host (Cheng et al., 2022). Pyridoxal, pyridoxine, and pyridoxamine are the three forms of vitamin B6, and pyridoxal 5′‐phosphate and 4‐pyridoxic acid are the two forms which are the key metabolites in the metabolic pathway of vitamin B6. Vitamin B6 can regulate the absorption of amino acids and fat metabolism and reduce the release of acetylcholine, further inhibiting gastrointestinal peristalsis and relieving visceral smooth muscle spasms to alleviate gastrointestinal discomfort (Mayengbam et al., 2020). The content of pyridoxamine decreased significantly in the model group compared to the blank group, and Dioscoreae Rhizoma starch did not play a regulatory role.

Bile acids metabolism involves many physiological processes in the host, and many diseases are related to abnormal bile acid metabolism (Jia et al., 2020). As an essential component of bile acids, bile acids exist in the intestinal and hepatic circulatory system, participate in fat metabolism, and play a protective role through recycling. Bile acids can stimulate intestinal epithelial cells and inhibit the reabsorption of water, electrolytes, etc. (Di Vincenzo et al., 2022). Bile acids can also regulate intestinal microbiotas to play a pharmacodynamic role (Kastl et al., 2022). Regulation of bile acids metabolism is an essential pathway for resistant starch from different plants to play a pharmacological role (Zhu et al., 2022). Our results showed that Dioscoreae Rhizoma starch alleviated the chronic diarrhea symptoms by regulating the metabolism of bile acids in rats with chronic diarrhea.

## 5 CONCLUSION

This study found that Dioscoreae Rhizoma starch could effectively alleviate diarrhea in chronic diarrhea rats. A 16S rRNA gene sequencing analysis and LC‐MS‐based metabolomics were performed to elucidate the potential mechanism of Dioscoreae Rhizoma starch on chronic diarrhea. The occurrence of chronic diarrhea is closely related to the disorder of intestinal microbiotas and endogenous metabolites. Based on the 16S rRNA gene sequencing analysis, we found that the intestinal microbiotas in chronic diarrhea rats had altered 17 different intestinal microbiotas at the genus level, and 11 kinds of intestinal microbiotas were regulated after being treated with Dioscoreae Rhizoma starch. Based on fecal metabolome analysis, we identified 46 potential biomarkers in chronic diarrhea rats, and Dioscoreae Rhizoma starch could regulate 23 biomarkers to alleviate diarrhea. These 23 endogenous metabolites were mainly involved in phenylalanine, tyrosine, and tryptophan biosynthesis; tyrosine metabolism; vitamin B6 metabolism; and purine metabolism. In conclusion, our study found that Dioscoreae Rhizoma starch could improve intestinal barrier integrity and alleviate intestinal inflammatory response in chronic diarrhea rats by improving intestinal microbiota and metabolomic features. Meanwhile, our study helps to further understand the mechanism of the Dioscoreae Rhizoma's efficacy and lays the foundation for further research on the Dioscoreae Rhizoma.

## AUTHOR CONTRIBUTIONS

**Qing Zhang:** Conceptualization (lead); data curation (lead); funding acquisition (equal); investigation (lead); validation (equal); writing – original draft (lead); writing – review and editing (lead). **Xu Zhang:** Investigation (equal); methodology (equal). **Qing Wang:** Investigation (equal); methodology (equal); writing – original draft (equal). **Suiqing Chen:** Funding acquisition (lead); project administration (lead); supervision (equal); writing – review and editing (equal).

## CONFLICT OF INTEREST STATEMENT

The authors declared that they have no conflicts of interest.

## Data Availability

The data that support the findings of this study are available on request from the corresponding author on reasonable request.
